# Homoepitaxial SrTiO_3_(111) Film with High Dielectric Performance and Atomically Well-Defined Surface

**DOI:** 10.1038/srep10634

**Published:** 2015-06-15

**Authors:** Yan Liang, Wentao Li, Shuyuan Zhang, Chaojing Lin, Chao Li, Yuan Yao, Yongqing Li, Hao Yang, Jiandong Guo

**Affiliations:** 1 College of Physics, Optoelectronics and Energy & Collaborative Innovation Center of Suzhou Nano Science and Technology, Soochow University, Suzhou 215006, China; 2 Beijing National Laboratory for Condensed Matter Physics & Institute of Physics, Chinese Academy of Sciences, Beijing 100190, China; 3 College of Science, Nanjing University of Aeronautics and Astronautics, Nanjing 211106, China; 4 Collaborative Innovation Center of Quantum Matter, Beijing 100871, China

## Abstract

The six-fold symmetry possessed by the (111) surfaces of perovskite oxides allows the epitaxial growth of novel quantum materials such as topological insulators. The dielectric SrTiO_3_(111) thin film is an ideal buffer layer, providing the readily tunability of charge density in gate-controlled structures. But the high-quality film growth is challenging due to its strong surface polarity as well as the difficulty of obtaining the chemical stoichiometry. Here we show that the layer-by-layer growth of homoepitaxial SrTiO_3_(111) thin films can be achieved in molecular beam epitaxy method by keeping the growing surface reconstructed. And the cation stoichiometry is optimized precisely with the reflective high energy electron diffraction as the feedback signal that changes sensitively to the variation of metal concentration during growth. With atomically well-defined surfaces, the SrTiO_3_(111) films show high dielectric performance with the charge density modulated in the range of 2 × 10^13^/cm^2^ with the back gate voltage lower than 0.2 V. Methods of further broadening the range are also discussed.

The low-dimensional transition metal oxides (TMOs) have a rich diversity of remarkable properties^1^,^2^,^3^, such as room-temperature ferroelectricity^4^, strong ferroelectric ferromagnetism^5^ and superconducting at the interface^6^, as well as strong polarization enhancement^7^. More importantly, their properties can be readily tuned by external fields^8^ so that the device functionalities are designed, just as in modern microelectronics based on conventional semiconductor interfaces^9^,^10^,^11^. The low-dimensional TMO structures based on perovskite ABO_3_(111) (A and B are cations) surfaces are particularly interesting. Along this direction, the crystal is stacked with alternative AO_3_ and B atomic layers. The so-called double perovskite A_2_BB’O_6_ in which the B/B’ cations are long-range ordered can be prepared as the [111] superlattice of AO_3_/B/AO_3_/B1^12^,^13^,^14^,^15^,^16^,^17^,^18^. Such an artificial material cannot be synthesized in the bulk form with the B/B’ cation doping. The dopant spatial distribution in bulk is dictated by thermodynamics and falls in randomness at the atomic scale in normal cases due to the close energies of different ordering configurations^12^. By growing the LaFeO_3_/LaCrO_3_(111) superlattice, the ordered double perovskite La_2_FeCrO_6_ film^15^,^19^,^20^ was successfully obtained and showed unique magnetic ordering, in contrast to the solid solution of LaFeO_3_ and LaCrO_3_ where the long-range ordering of Fe/Cr distribution is missing.

The six-fold symmetry possessed by perovskite ABO_3_(111) also provides the opportunity to achieve novel quantum states. It was predicted that the double-layered ABO_3_(111) might be promising candidate of new topological insulators (TIs)^21^,^22^,^23^,^24^. Comparing with conventional TIs whose electric properties are dominated by *s* and *p* orbitals, TMO-based topological materials would be more flexible to control since *d* electrons with strong correlation contribute significantly to their electronic properties. The honeycomb lattice formed by the two trigonal sublattices on the top two layers of the perovskite (111) surface could reduce the symmetry of crystal field and introduce additional level splitting of *d* orbitals. Xiao *et al.* found that LaAuO_3_(111) has a large topological nontrivial band gap and could realize quantum spin Hall effect at room temperature^23^.

Strontium titanate, SrTiO_3_ is a typical perovskite oxide with very high dielectric performance. Its (111) surface matches the lattices of many novel quantum materials with hexagonal in-plane structure. Therefore SrTiO_3_(111) with an atomically well-defined surface not only serves as an epitaxial growth template, but also can be used as the insulating layer for the modulation of carriers density by gate electric field. Chen *et al.* reported the control of charge density of Bi_2_Se_3_ on SrTiO_3_(111) in the range of 2 × 10^13^/cm^2^ by applying the back gate voltage of 150 V^25^. Apparently the SrTiO_3_(111) thin film as a dielectric buffer layer on a metallic substrate would have effectively reduced the gate voltage required for carriers modulation thus making the structure convenient for device applications. However, due to the surface polarity of the ionic crystal^26^, it is essentially challenging to realize the high-quality layer-by-layer growth of SrTiO^3^(111) films. In the current work, we achieve the growth of high-quality homoepitaxial SrTiO_3_(111) films on Nb-doped metallic substrates by molecular beam epitaxy (MBE) method. Reconstructions are formed on the substrate surface and maintained all through the growth to compensate the surface polarity, enabling the layer-by-layer growth with atomically well-defined film surface. And with the monitor of reflective high-energy electron diffraction (RHEED) on the microstructure corresponding to different reconstruction phases of the growing surface, the deposition rates of metal sources are precisely controlled to realize the cation stoichiometry. The films show high dielectric performance. At 6 K, a 50 nm-thick film allows the carriers density modulation in the order of 2 × 10^13^/cm^2^ with the gate voltage less than 0.2 V. We present an ideal platform for the design and growth of low-dimensional quantum structures with easy and reliable tunability of the carriers.

## Experiment

The experiments were carried out in oxide MBE system equipped with a scanning tunneling microscope (STM). The base pressure was better than 1 × 10^−10^ mbar. The Nb-doped (0.7 wt.%) SrTiO_3_ (111) substrate was heated by passing a direct current and the temperature was measured with an infrared pyrometer. The monophased (4 × 4) reconstructed surface was prepared by Ar^+^sputtering (1 kV / 10 *μ*A) for 12 minutes followed by annealing in oxygen with the pressure of 3 × 10^−6^ mbar at 1000 °C for 1 hour. Then the (6 × 6) reconstruction was formed by depositing 1.1 monolayer [ML, 1 ML = 3.8 × 10^14^ atom/cm^2^, corresponding to the cation density on the bulk truncated SrTiO_3_(111) surface] Ti atoms on (4 × 4) at 1000 °C with oxygen pressure of 3 × 10^−6^ mbar^27^. High purity (99.999%) Sr and Ti were evaporated by a low-temperature and a high-temperature effusion cells, respectively. The depositing rates of the sources were calibrated on SrTiO_3_(110) surface according to the reconstruction evolution behavior upon the surface metal cation concentration^28^. By setting the temperature of Sr and Ti sources at 420 °C and 1450 °C, respectively, the same flux rates of 0.4 ML/min from both sources were obtained. SrTiO_3_(111) film was grown by co-evaporating Sr and Ti under an oxygen pressure of 3 × 10^−6^ mbar with the substrate temperature of 1000 °C, followed by *in situ* annealing at 400 °C with oxygen pressure of 5 × 10^−3^ mbar for 3 hours. The cross-sectional microstructure of the film was characterized by high-resolution transmission electron microscopy (HRTEM) (FEI Tecnai G2 F20). The dielectric performance of the film was measured by a microampere meter (Agilent B2901A) that provides the gate voltage, and a lock-in amplifier (SR830) that obtains the capacitive reactance in the measurement circuit with the test signal of 20.14 Hz/1 mV at 6 K.

## Results and Discussions

Although the deposition rates of Sr and Ti sources have been calibrated on SrTiO_3_(110) surface^29^, further precise tuning is necessary for the MBE growth of SrTiO_3_(111) films due to the possibly different adhesion coefficients of metals. It has been reported that the SrTiO_3_(111) surface shows a series of reconstructions that evolve upon the cation concentration, *e.g.*, a monophased (4 × 4) can be changed into (6 × 6) by depositing ~1.1 ML Ti on the surface and the transformation is totally reversible by depositing the same amount of Sr on (6 × 6)^27^. More importantly, the change between different microstructures corresponding to those reconstruction phases can be monitored by RHEED even at high temperature in real-time during the metal evaporation. This allows us to precisely synchronize the deposition rate of Sr and Ti during the MBE growth of the SrTiO_3_(111) films to optimize the cation stoichiometry. In the current work, we prepare a monophased SrTiO_3_(111)-(6 × 6) substrate surface and basically keep it unchanged all through the film homoepitaxial growth by adjusting the temperatures of Sr and Ti sources with RHEED patterns as the feedback control signal.

**Figure 1:**
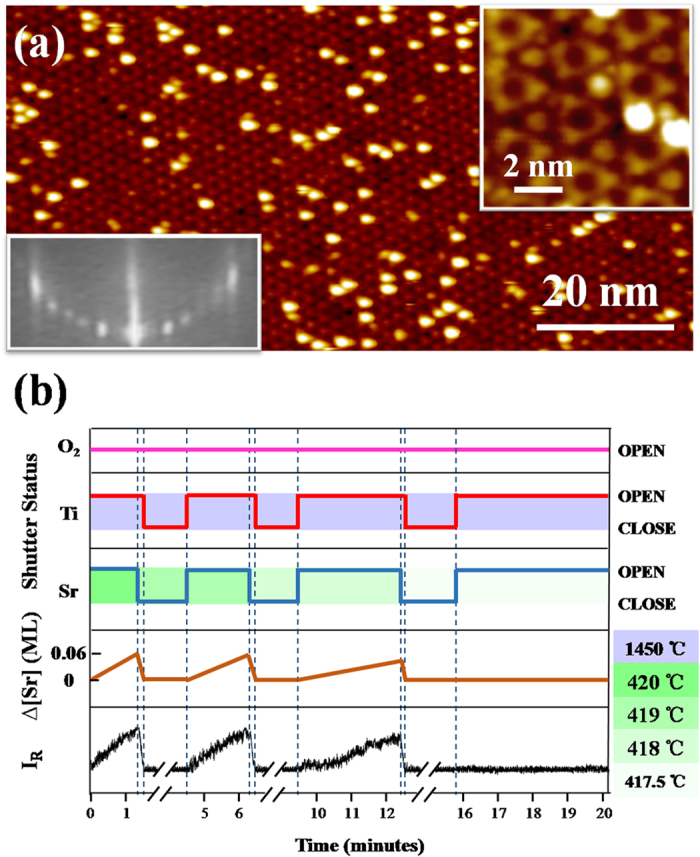
(**a**) The STM image (+2.5 V/50 pA) of the (6 × 6)-reconstructed SrTiO_3_(111) substrate surface. The right inset shows the zoom-in features and the left inset shows RHEED patterns of the corresponding surface. (**b**) Procedures of adjusting the source temperature for the optimization of film cation stoichiometry. The change of ΔSr is schematically shown while the corresponding intensity of RHEED (01) spot I_*R*_ is presented in the bottom panel.

Figure 1 (a) shows the STM image of the (6 × 6)-reconstructed substrate surface. The corresponding sharp RHEED patterns of the surface also indicate the high quality of the long-range ordered surface. The SrTiO_3_(111) film is deposited with the substrate temperature at 1000 °C and oxygen pressure at 3 × 10^−6^ mbar. And the temperatures of Sr and Ti sources are initially set at 420 °C and 1450 °C, respectively. The intensity increase of RHEED (01) diffraction spot can be sensitively observed [see the bottom panel of Fig. 1 (b)]. And after 15-minutes growth, “ 4 × “ fractional spots appear in RHEED indicating that the Sr rate is higher than Ti. Knowing this relationship between the RHEED intensity (I_*R*_) and the Sr concentration relative to that on the monophased (6 × 6) surface (ΔSr), we immediately (after 86-seconds growth) recover ΔSr to 0 by closing the shutter of Sr source and evaporating Ti only (for 9 seconds) until I_*R*_ drops back to the value as on the initial substrate surface. It can be estimated that during the 86-seconds growth, Sr deposits more than Ti by ~0.06 ML. Then both shutters of Sr and Ti sources are closed and the temperature of Sr source is reduced to 419 °C. Coevaporating Sr and Ti again, I_*R*_ still increases with time although the slope is lowered. The above procedures are repeated until I_*R*_ keeps constant for a long time [*e.g.*, 4 minutes as shown in Fig. 1 (b)]. With the temperatures set at 417.5 °C and 1450 °C for Sr and Ti sources, respectively, they provide precisely the same evaporation rates.

**Figure 2:**
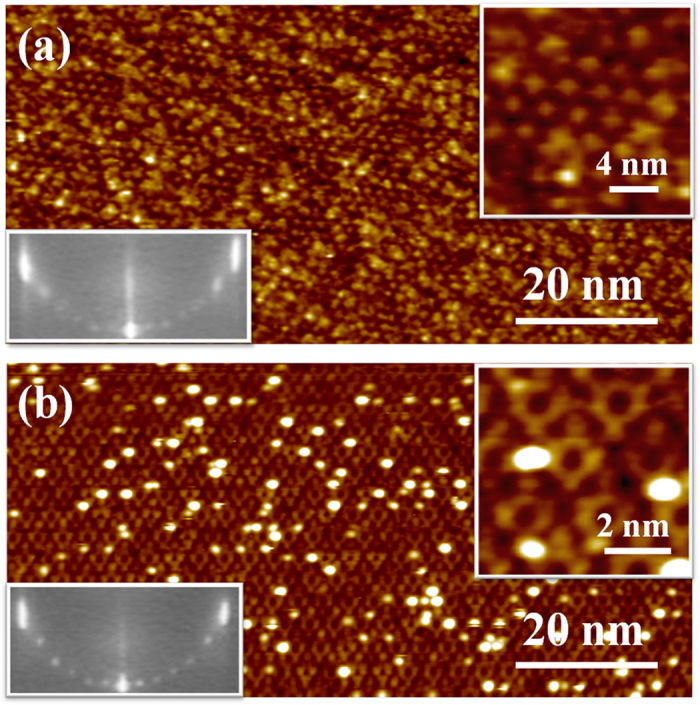
(**a**) The STM image (+2.3 V/50 pA) of the as-grown surface of the 30 nm film. (**b**) The STM image (+2.2 V/50 pA) of the film surface after depositing 0.12 ML Ti onto the surface shown in (**a**). The right insets show the zoom-in features and the left insets show RHEED patterns of the corresponding surfaces, respectively.

Growing for 340 minutes, a 30-nm thick SrTiO_3_(111) film is obtained. Figure 2 (a) shows the STM image of the as-grown surface. Although the (6 × 6) periodicity is still visible, the surface is covered by some disordered adsorbates. And the fractional RHEED patterns become fuzzy comparing to the initial monophased (6 × 6) substrate surface. We find that the quality of film surface can be completely recovered by depositing 0.12 ML Ti, exposing the well-ordered (6 × 6) lattice with negligible amount of disordered adsorbates [Fig. 2 (b)]. Considering Sr and Ti in equivalent amount induce the change of surface microstructure in the reverse way^27^, it is indicated that ΔSr increases to 0.12 ML after 340-minute growth. Therefore the deviation from the cation stoichiometry is less than 0.1% in the film. Actually, if we extend the calibration time as illustrated in Fig. 1 (b), the precision of cation stoichiometry could be further improved since the change of I_*R*_ will accumulate to be sensitive for detection.

**Figure 3:**
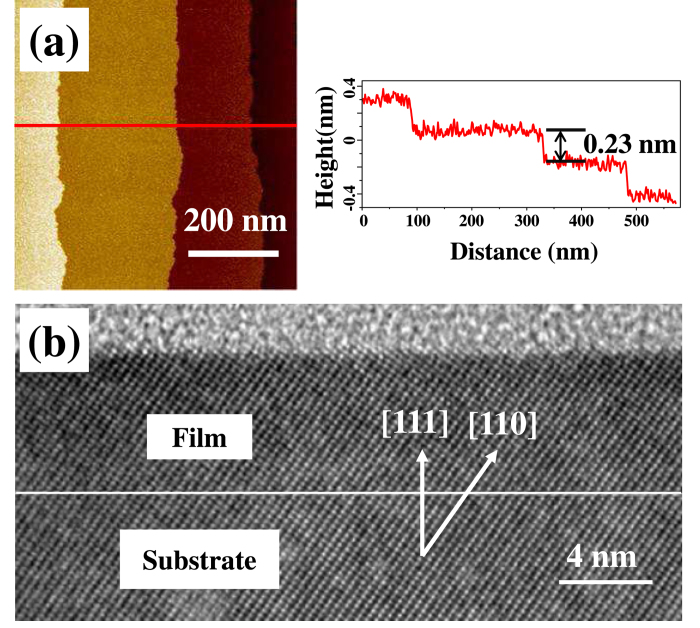
(**a**) The wide-range STM image (+2.3 V/50 pA) of the 30 nm SrTiO_3_(111) film surface. The height profile along the red line (the left panel) is shown in the right panel. (**b**) The cross-sectional HRTEM image of a 6-nm-thick film.

Figure 3 (a) shows the large-scale STM image of the 30-nm film surface. The statistics over several images indicate the average terrace width of ~150 nm, significantly broadened relative to the substrate (<100 nm). And the surface topography shows the step height of single-unit-cell (0.23 nm). These are the signature of the high-quality layer-by-layer growth of the SrTiO_3_(111) film, although the oscillation of RHEED intensity during film growth, typically associating the layer-by-layer growth, is not observed. The case is similar to the layer-by-layer growth of SrTiO_3_(110) film when the RHEED incident angle was in a certain range making it to be sensitive to the surface microstructure rather than the atomic layer roughness^30^. The cross-sectional HRTEM also confirms the nice crystallinity of the (111) film with indistinguishable interface of the homoepitaxial structure, as shown in Fig. 3 (b). We cap the film *in situ* with a layer of amorphous Si to mark the surface position in HRTEM. And the interface position can be estimated by counting the atomic layers from the surface since the growth thickness is known.

Keeping the growing surface reconstructed is the key to obtain the high-quality SrTiO_3_(111) film. Firstly, the microstructure evolves upon the amount of Sr/Ti deposited on the surface, allowing the sensitive monitoring by RHEED. Secondly, the surface polarity is effectively compensated by the reconstruction, facilitating the layer-by-layer film growth to obtain the atomically well-defined surface. Therefore the substrate temperature should be relatively high (1000 °C in the current work) to provide enough energy to deposited metal atoms diffusing on the surface to form the long-range ordered reconstruction promptly during the film growth. The reconstruction does not have to be limited to the (6 × 6) phase, as long as it is in the reversible range^27^. Tuning the substrate preparation parameters, *i.e.*, the sputtering dosage or the initial Sr/Ti deposition amount, different reconstructions can be obtained for the homoepitaxial growth. Once the relationship between RHEED intensity and surface Sr/Ti concentration is established, the feedback signal will be obtained to optimize the cation stoichiometry precisely.

**Figure 4:**
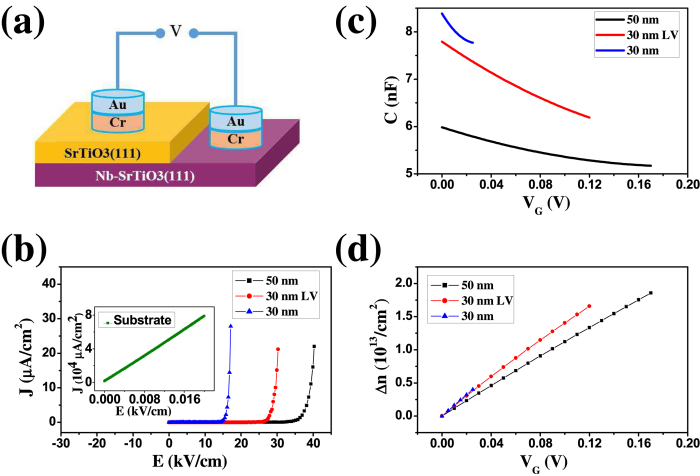
(**a**) The schematic drawing of the Au/Cr/SrTiO_3_/Nb-SrTiO_3_ capacitor structure. (**b**) The leakage current density at different electric field, (**c**) the capacitance at different bias and (d) the charge density modulation range with different gate voltage measured at 6 K, respectively.

The SrTiO_3_(111) thin film with atomically well-defined surface can be used not only as a epitaxial template of many novel quantum materials, but also as an ideal insulating layer of gate structure for the tuning of carries density. We fabricate the back gate structure by evaporating 3-nm-Cr / 30-nm-Au electrodes with the size of 200 *μ*m in diameter on the film, while the conducting Nb-doped substrate with high carrier density serves as the other electrode, as schematically shown in Fig. 4 (a). On the Nb-doped SrTiO_3_ substrate, a Au/Cr/SrTiO_3_/Nb-SrTiO_3_/Cr/Au capacitor is formed to characterize the dielectric performance of the SrTiO_3_ film. For the 30-nm thick film at 6 K, the leakage current density (J) keeps extremely low (<1 *μ*A/cm^2^) until the electric field (E) reaches 14 kV/cm (the breakdown field, corresponding applied voltage is 45 mV), and above the breakdown field J increases exponentially, as plotted in Fig. 4 (b). The capacitance-voltage (C-V) measurement indicates the capacitance of 8.4 nF at 0 V, which decreases to 7.8 nF at 25 mV. The relative dielectric constant *ε* is calculated as 910 at 0 V with a slight decay when a bias applied. Importantly the modulation amplitude of carriers density at the electrode with a gate voltage V_*G*_ [Δ*n*(V_*G*_)] can be determined by 
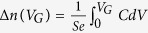
 , where *S* is the area of the electrode and *e* is the unit charge. Figure 4 (d) shows the dependence of Δ*n* on V_*G*_ - Δ*n* is ~4.5 × 10^12^/cm^2^ with the gate voltage as low as 25 mV. It has been reported that dopants in SrTiO_3_ might be redistributed due to high-temperature annealing^31^. To exclude the substrate effect on the measurement of dielectric performance of the homoepitaxial film, we anneal the Nb-doped substrate in oxygen with the pressure of 3 × 10^−6^ mbar at 1000 °C for 6 hours followed by annealing in oxygen with the pressure of 5 × 10^−3^ mbar at 400 °C for 3 hours (the same annealing as for the substrate for film growth). As shown in the inset of Fig. 4 (b), a purely resistive behavior with a high conductivity at 6 K was detected, distinct from the dielectric film. It is evident that the substrate has negligible influence on the quantitative determination of the charge modulation range of the homoepitaxial film.

In order to improve Δ*n* of SrTiO_3_(111) thin film on Nb-doped substrate, we reduce the amount of residual oxygen vacancies (V_*O*_s) in the film by annealing at 400 °C in a tube furnace with oxygen flow at ~1 bar for 16 hours. As shown in Fig. 4 (b), the insulating performance of the 30-nm film (with a low oxygen vacancy density, LV) is effectively improved, resulting in the increase of breakdown field to 26 kV/cm. And Δ*n* can be tuned in the range of 1.7 × 10^13^/cm^2^ by V_*G*_ lower than 120 mV [Fig. 4 (d)]. It should be noted that, due to the exposure of film surface to air and the following contaminations at high temperature, the *ex situ* annealing leads to the formation of an amorphous layer on the film, which seriously destroys the atomically well-defined surface as characterized by STM. Additionally the amorphous layer has a lower dielectric constant than SrTiO_3_ and introduces a small serial capacitor to the measured back gate structure. As the consequence, both of the total capacitance and dielectric constant are deteriorated in an uncontrolled way.

Alternatively, Δ*n* can be enhanced by increasing the film thickness. In a thick insulating layer, defects of holes are difficult to line up to form the conducting channels. Therefore the breakdown field of a 50 nm thick film is significantly increased to 33 kV/cm [Fig. 4 (b)]. The relative dielectric constant also increases to 1000 (at 0 V), comparable with the reported values of SrTiO_3_(001) films with the thickness of several hundreds of nanometers^32^,^33^,^34^. At V_*G*_ = 170 mV, the maximal Δ*n* reaches 1.9 × 10^13^/cm^2^. The estimation of the dieletric performance of even thicker films can be made by a first-order appoximation. For example, the breakdown field of the 100-nm film could be expected as ~95 kV/cm, with *ε* of about 1300 and the maximal Δ*n* of 6 × 10^13^/cm^2^ (V_*G*_ = 950 mV). In such a way, the atomically well-defined surface of SrTiO_3_(111) film is maintained, facilitating the following epitaxial growth of other quantum materials.

## Conclusions

We grow high-quality homoepitaxial SrTiO_3_(111) thin film on Nb-doped substrate by MBE. The growing surface is kept reconstructed to compensate the surface polarity and thus guarantee the layer-by-layer growth mode. And determined by the surface cation concentration, the formation of different reconstruction phases can be monitored by RHEED, providing the sensitive feedback signal for the optimization of chemical stoichiometry in real-time during film growth. Besides the atomically well-defined surface microstructure, the films show high dielectric performance that allow the modulation of carriers density in the range of 2 × 10^13^/cm^2^ with the back gate voltage lower than 0.2 V. The film provides an ideal platform for the epitaxial growth of novel quantum materials as well as the control of electronic properties in their device structures.

## Additional Information

**How to cite this article**: Liang, Y. *et al.* Homoepitaxial SrTiO3(111) Film with High Dielectric Performance and Atomically Well-Defined Surface. *Sci. Rep.* **5**, 10634; doi: 10.1038/srep10634 (2015).
